# Adaptive Mesh Strategy for Efficient Use of Interface Elements in a 3D Probabilistic Explicit Cracking Model for Concrete

**DOI:** 10.3390/ma17153786

**Published:** 2024-08-01

**Authors:** Magno T. Mota, Pierre Rossi, Eduardo M. R. Fairbairn, Fernando L. B. Ribeiro, Jean-Louis Tailhan, Henrique C. C. Andrade

**Affiliations:** 1 Civil Engineering Program, COPPE, Universidade Federal do Rio de Janeiro (UFRJ), Rio de Janeiro 21941-598, Brazil; eduardo@coc.ufrj.br (E.M.R.F.); fernando@coc.ufrj.br (F.L.B.R.); henriqueconde@coc.ufrj.br (H.C.C.A.); 2 Department of Materials and Structures, Université Gustave Eiffel, 13015 Marseille, France; jean-louis.tailhan@univ-eiffel.fr

**Keywords:** mesh adaptivity, probabilistic explicit cracking model, concrete, finite element method, scale effect

## Abstract

In this paper, the development of a 3D adaptive probabilistic explicit cracking model for concrete is reported. The contribution offered herein consists in a new adaptive mesh strategy designed to optimize the use of interface elements in probabilistic explicit cracking models. The proposed adaptive mesh procedure is markedly different from other strategies found in the literature, since it takes into account possible influences on the redistribution of stresses after cracking and can also be applied to purely deterministic cracking models. The process of obtaining the most appropriate adaptive mesh procedure involved the development and evaluation of three different adaptivity strategies. Two of these adaptivity strategies were shown to be inappropriate due to issues related to stress redistribution after cracking. The validation results demonstrate that the developed adaptive probabilistic model is capable of predicting the scale effect at a level similar to that experimentally observed, considering the tensile failure of plain concrete specimens. The results also show that different softening levels can be obtained. The proposed adaptive mesh strategy proved to be advantageous, being able to promote significant reductions in the simulation time in comparison with the classical strategy commonly used in probabilistic explicit cracking models.

## 1. Introduction

Several studies have been conducted using probabilistic numerical models that deal directly with the heterogeneity of concrete [1,2,3,4,5,6,7,8,9,10,11,12,13]. With the statistical distribution of mechanical properties and possible use of fracture mechanics concepts, the failure process of structures can be analyzed in a more realistic and comprehensive way. In such models, manifestations of the scale effect, which involve the variation in global mechanical property evaluations with the change in the structure size, and softening behavior (gradual loss of load-bearing capacity) are intrinsic consequences, and the statistical treatment of global results is enabled by the Monte Carlo method.

One of the probabilistic approaches directed to the modeling of concrete behavior is the explicit cracking approach originally proposed by Rossi and Richer [1]. This approach is based on the finite element method (FEM), being mainly characterized by the use of zero-thickness interface elements to which the following purposes are assigned: to receive mechanical properties distributed randomly and to represent the material discontinuities (failure planes) explicitly. The models developed from this idea have advantages in situations of crack opening assessment, which can be performed directly, in addition to favoring the implementation of strategies to deal directly with phenomena that occur during the fracture process, such as the contact between crack surfaces.

The development of 3D probabilistic explicit cracking models remains at the initial stage, with considerable limitations imposed by their high computational cost. The inclusion of interface elements in the mesh significantly increases the number of nodes and, consequently, the execution time of the analyses, especially when quadratic elements are employed. It is important to think of strategies to deal with this issue, since three-dimensional analyses are indispensable in some cases, with the cracking process tending to be naturally three-dimensional. In such cases, for more realistic and comprehensive modeling, the fracture mechanisms cannot be neglected in any dimension. For safety and durability reasons, an important potential application of 3D models concerns, for example, the study of thermal cracking of massive concrete structures, such as dams and nuclear power plants. The computational cost issue can be handled by using a finite element code designed for high performance, proper data structures [14,15], and mesh adaptivity and computational parallelization strategies.

Mota et al. [11] developed a 3D probabilistic explicit cracking model capable of reproducing different levels of softening behavior and predicting the scale effect in tension with an intensity similar to that experimentally observed. This is of great importance, since the scale effect and softening behavior occur naturally for quasi-brittle materials such as concrete and strongly influence the fracture process of these materials. The mentioned model was implemented following the classical procedure of using interface elements between all the solid elements since the beginning of the analysis. Although the code was written on a high-performance FEM platform and parallelization strategies were employed, the computational cost can still be considerably reduced by adopting a mesh adaptivity strategy for the controlled use of interface elements.

As the use of interface elements is a strong time-consuming factor, an adaptivity strategy for inserting interface elements only in the regions of the mesh where they are indispensable is an important alternative to speed up the analyses. There are studies performed with adaptive mesh procedures in accordance with this idea [16,17,18,19,20,21,22,23], but these works do not encompass probabilistic cracking models and almost all of them [16,17,18,19,20,21,22] do not take into account the influence of the proposed adaptivity strategy on the redistribution of stresses after cracking. Furthermore, the adaptivity strategies proposed by Zhan and Meschke [22] and Rodrigues et al. [23] consider a different type of interface element (interface solid element with a high aspect ratio). Thus, the adaptive mesh strategies developed in all the mentioned studies [16,17,18,19,20,21,22,23] cannot be applied to the probabilistic explicit cracking approach, so a new strategy is necessary. An adaptivity procedure appropriate for probabilistic explicit cracking models can prove to be very efficient, for example, in simulations in which the structural failure occurs due to concentrated cracking in delimited regions of the mesh.

In view of the foregoing considerations, this work reports the development of a 3D adaptive probabilistic cracking model based on the explicit cracking approach proposed by Rossi and Richer [1]. The model in question represents an improvement of the one implemented by Mota et al. [11]. The contribution offered herein consists in an adaptive mesh strategy designed to optimize the use of interface elements in probabilistic explicit cracking models, taking into account possible influences on the redistribution of stresses after cracking. This strategy is markedly different from the strategies found in the literature [16,17,18,19,20,21,22,23] and can also be applied to purely deterministic cracking models that employ interface elements.

## 2. Empirical Relations for Scale Effect

Rossi et al. [24], by means of an extensive experimental investigation, obtained analytical expressions that define correlations between concrete heterogeneity and scale effects, contributing to the validation of probabilistic models. These expressions depend on the $V_{T}/V_{A}$ ratio, which is a parameter of heterogeneity, and on the compressive strength ($f_{c}$). The parameter $V_{T}$ is the total volume of the specimen, and $V_{A}$ is the volume of the coarsest aggregate, modeled by a sphere. The expressions are applicable to any concrete, except fiber-reinforced and lightweight concretes, with an $f_{c}$ ranging from 35 MPa to 130 MPa and with the $V_{T}/V_{A}$ ratio varying from 10 to 10,000 in order of magnitude. For the mean value of tensile strength or mean tensile strength ($f_{t}^{mean}$) and the coefficient of variation (CV), which is a measure of dispersion defined as the ratio between the standard deviation (SD) and the mean tensile strength, Rossi et al. [24] obtained the following relations:

(1) $$f_{t}^{mean}=a{\left(V_{T}/V_{A}\right)}^{-\gamma }$$

(2) $$CV=A{\left(V_{T}/V_{A}\right)}^{-\beta }$$

in which a=6.5 MPa, A=0.35, and the values of γ and β are defined by

(3) $$\gamma =0.25-3.6\times 10^{-3}\frac{f_{c}}{c_{1}}+1.3\times 10^{-5}{\left(\frac{f_{c}}{c_{1}}\right)}^{2}$$

(4) $$\beta =4.5\times 10^{-2}+4.5\times 10^{-3}\frac{f_{c}}{c_{1}}-1.8\times 10^{-5}{\left(\frac{f_{c}}{c_{1}}\right)}^{2}$$

with $c_{1}=1$ MPa.

**Figure 1:**
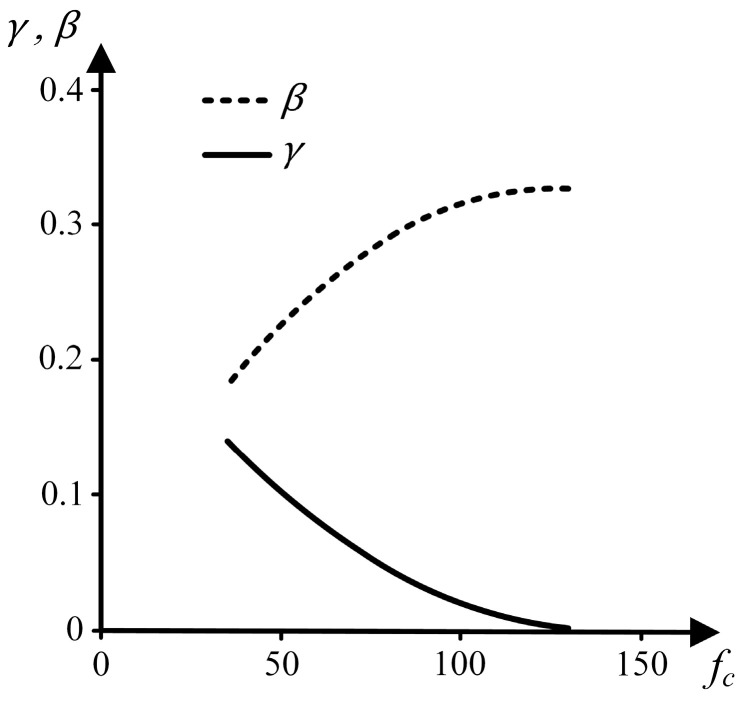
Diagrams of γ and β versus $f_{c}$ (parameters related to Equations (1)–(4)).

Figure 1 portrays the diagrams of γ and β versus $f_{c}$. With the aid of these diagrams, it is possible to note that, with γ and β being always positive, the higher the $V_{T}/V_{A}$ ratio in Equations (1) and (2), the lower the mean tensile strength and the coefficient of variation. Figure 1 also shows that, with the compressive strength being a quality indicator of the cement paste, when $f_{c}$ increases, γ decreases, leading to higher values of mean tensile strength. On the other hand, higher values of $f_{c}$ result in higher values for β and, consequently, in lower values for CV.

According to Rossi et al. [24], the tensile strength dispersion is occasioned by the material heterogeneity, being directly associated with the scale effect. Small values of CV, obtained either by increasing the $V_{T}/V_{A}$ ratio or by increasing the value of $f_{c}$, represent a low influence of the heterogeneity on the results and, consequently, a low-intensity scale effect.

## 3. 3D Adaptive Probabilistic Explicit Cracking Model

**Figure 2:**
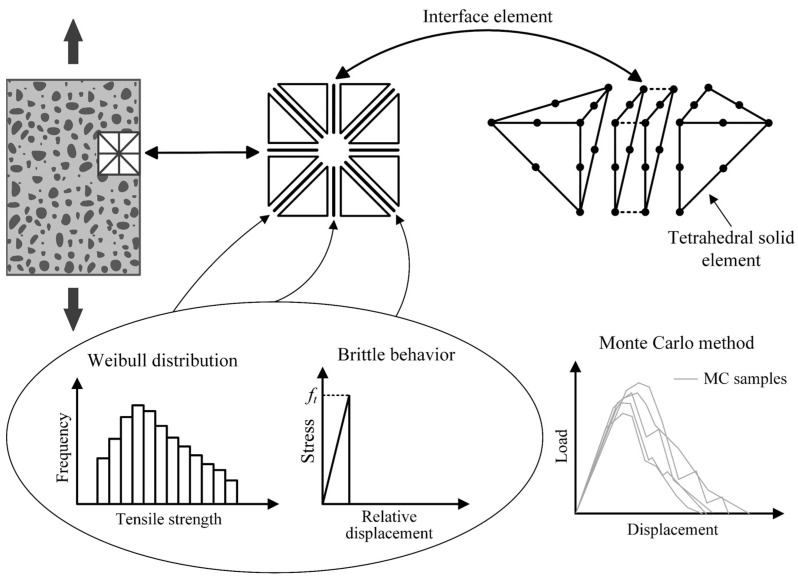
Important features of the probabilistic cracking model implemented by Mota et al. [11]: use of tetrahedral solid elements that remain undamaged; use of interface elements following a brittle behavior to represent cracking; local random distribution of tensile strength values; and Monte Carlo method for structural responses.

Figure 2 outlines the 3D probabilistic explicit cracking model without mesh adaptivity developed by Mota et al. [11], which is the basis of the present work, including for comparison purposes. The main characteristics of this model are described as follows:

- The three-dimensional treatment is basically given by the use of tetrahedral solid elements of 10 nodes;
- Zero-thickness interface elements of 6 + 6 nodes are previously inserted between all the solid elements in accordance with the classical method of mesh formation;
- Quadratic interpolation functions are used in the elements to obtain results with a higher precision;
- The heterogeneous and probabilistic character is established by the random assignment of tensile strength values to the interface elements according to the Weibull distribution. Thus, concrete is being defined as a heterogeneous material, since there is no tensile strength variation for a homogenous material. Weibull’s statistical law was chosen for two reasons: (1) it was developed for brittle materials, which can be associated with the fact that a brittle law is considered for the material locally; (2) negative values of random properties are not possible;
- Solid elements have a linear elastic behavior;
- Interface elements follow a sufficiently rigid and brittle behavior under tension, which is naturally associated with the Rankine failure criterion, resulting in local geometric and material discontinuity when the tensile strength of a given interface element is reached, causing the occurrence of a cracked interface element. Thus, during the computational analysis, there are local nonlinearities that generate nonlinearity in the global response;
- Structural softening is naturally obtained according to the distribution of tensile strength among the interface elements;
- When there is contact between the faces of cracked interface elements (contact problem locally), there is a reactivation of stiffness in the interface elements to take into account compression and friction effects;
- The acquisition of structural responses occurs by means of the Monte Carlo method.

The constitutive relation for the interface element can be basically written in the form of the following damage-type model [11]:

(5) $$\left\{\sigma \right\}=\left(1-\delta \right)\left[k\right]\left\{w\right\}=\left(1-\delta \right)\left[\begin{array}{ccc} k_{11} & 0 & 0\\ 0 & k_{22} & 0\\ 0 & 0 & k_{33} \end{array}\right]\left\{\begin{array}{c} w_{t_{1}}\\ w_{t_{2}}\\ w_{n} \end{array}\right\}$$

in which δ is the damage parameter, $k_{11}$ and $k_{22}$ are penalty parameters associated with the tangential stiffness, $k_{33}$ is related to the normal stiffness, $w_{t1}$ and $w_{t2}$ are tangential relative displacements, and $w_{n}$ is the normal relative displacement. In order to obtain a brittle behavior, it is assumed that δ=0 for the intact interface element, and δ=1 when the tensile stress reaches the tensile strength (occurrence of a cracked interface element).

The friction between crack surfaces, which are represented by faces of cracked interface elements, is taken into account by means of the Mohr–Coulomb criterion [25] without cohesion:

(6) $$\left|\tau \right|-\sigma_{c}\tan\phi \leq 0$$

where τ is the shear stress or, more specifically, the acting friction stress in the cracked interface element; $\sigma_{c}$ is the absolute value of the normal compressive stress; and ϕ is the angle of friction. A more detailed description of the friction model is provided by Mota et al. [12].

The next subsections describe the implementation of mesh adaptivity in the model portrayed in Figure 2. For convenience, the regions where interface elements can be inserted are referred to as interfacial regions of solid elements or simply interfacial regions.

### 3.1. Generation and Distribution of Random Tensile Strength

For the Weibull distribution [26], the cumulative distribution function and the inverse cumulative distribution function related to a random variable x can be written respectively as

(7) $$F(x,b,c)=1-e^{-{\left(\frac{x}{c}\right)}^{b}}=y$$

(8) $$G(y,b,c)=c{\left(-\ln\left(1-y\right)\right)}^{\frac{1}{b}}=x$$

in which x≥0, with this variable corresponding to the tensile strength in the present paper; b is the shape parameter, associated with the coefficient of variation; and c is the scale parameter, directly related to the mean value of x.

The generation of random values of tensile strength is based on the inverse transformation method or uniform probability transformation technique [27]. These values are generated and distributed to interfacial regions of solid elements according to Algorithm A1 (Appendix A). If the values of b and c are fixed, a different Monte Carlo sample for the same material will be obtained each time Algorithm A1 is used.

### 3.2. Adaptive Mesh Strategies for Efficient Use of Interface Elements

**Figure 3:**
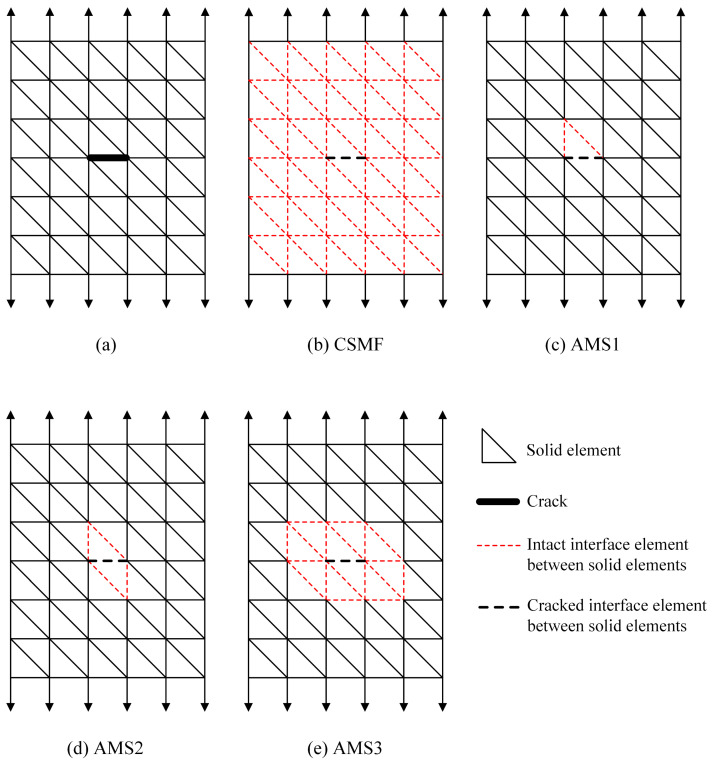
(**a**) Mesh without interface elements with a crack detected in an interfacial region of solid elements; (**b**) classical strategy of mesh formation for probabilistic explicit cracking models—CSMF; (**c**) Adaptive Mesh Strategy 1—AMS1; (**d**) AMS2; (**e**) AMS3.

Regarding finite element meshes for mechanical problems, a relevant challenge is how to represent the crack explicitly. For a mesh composed only of solid elements, a crack at an interfacial region of the elements, as indicated in Figure 3a, can be detected relatively simply through the calculation of nodal stresses in the region. However, the incorporation of the geometric discontinuity generated by the crack requires strategies of greater complexity.

**Figure 4:**
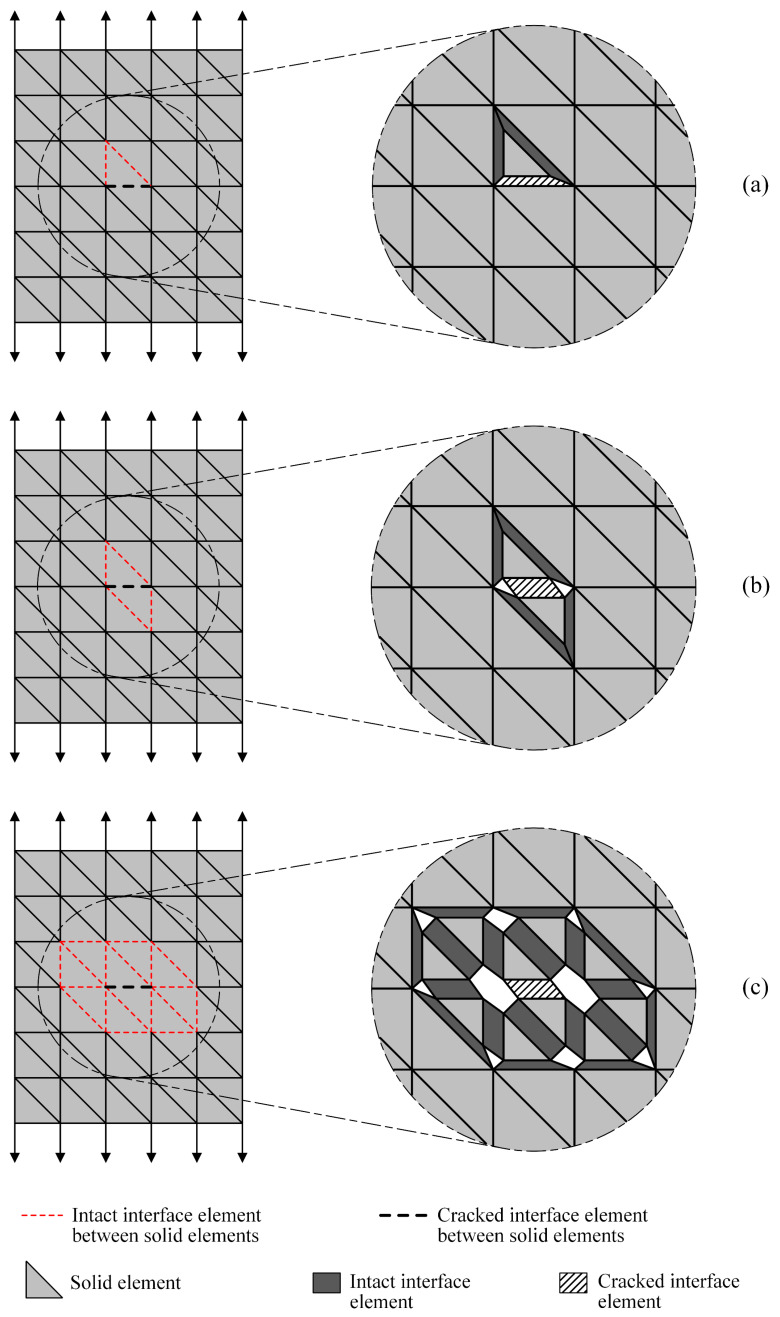
Crack zone in detail for the three considered adaptive mesh strategies: (**a**) AMS1; (**b**) AMS2; (**c**) AMS3.

Figure 3b illustrates the classical strategy adopted in probabilistic explicit cracking models to deal with the appearance of cracks, while Figure 3c–e present the three adaptive mesh strategies developed and evaluated in the context of this work. Figure 4 provides more details about the adaptivity options, with the solid elements being displayed in a reduced size in the crack zone so that the interface elements are highlighted. Two dimensional representations are used for simplification purposes.

In the here-called Classical Strategy of Mesh Formation (CSMF), shown in Figure 3b, the mesh is initially prepared with interface elements inserted between all the solid elements. Thus, the crack observed in Figure 3a is detected directly in the interface element, which produces a geometric discontinuity when its rigidity is nullified, instantly generating stress redistribution around the crack. However, this strategy causes a significant increase in the number of nodes from the beginning of the analysis, with interface elements being used even in regions where these elements may be unnecessary.

The three previously mentioned mesh adaptivity strategies were developed so that interface elements could be employed only in regions where they are indispensable. In these strategies, the mesh is initially composed only of solid elements, with interface elements being used during the analysis as cracking occurs. Consequently, after the beginning of the analysis, there may or may not be interface elements in the interfacial regions of the solid elements. When a crack is detected at an interfacial region with no interface element, a cracked interface element is inserted in this region. However, for mesh conformity, it is also necessary to place intact interface elements in the vicinity of the cracked interface element.

In the first mesh adaptivity strategy, referred to as Adaptive Mesh Strategy 1 (AMS1) and portrayed in Figure 3c and Figure 4a, intact interface elements are inserted around the solid element that provides the top face of the cracked interface element. As the choice for the solid element related to the top face was purely arbitrary, one could opt to use intact interface elements around the solid element associated with the base face of the cracked interface element instead. In a 2D mesh composed of triangular elements, this strategy would entail the inclusion of at most two intact interface elements for each detected crack. In a 3D configuration with tetrahedral solid elements, at most three intact interface elements are included for each new crack.

In Adaptive Mesh Strategy 2 (AMS2), depicted in Figure 3d and Figure 4b, intact interface elements are placed around the solid elements that provide the base and top faces of the cracked interface element. In this way, for each new crack, a maximum of four intact interface elements would be inserted in a 2D case with triangular elements; on the other hand, in 3D meshes of tetrahedrons, a maximum of six intact interface elements can be added.

In Adaptive Mesh Strategy 3 (AMS3), which is represented in Figure 3e and Figure 4c, intact interface elements are placed around all the solid elements in the vicinity of the cracked interface element. In this case, for each new crack, the maximum number of intact interface elements that can be inserted depends on the level of mesh discretization in the crack zone.

Details of the computational implementation of AMS1, AMS2 and AMS3, such as utilized arrays, explained synthesized algorithms, and further information about the insertion of cracked and intact interface elements in the mesh, are described in Appendix B.

### 3.3. Computational Program for Finite Element Analyses

The finite element code, written in Fortran language, was implemented in a high-performance computational platform developed by Ribeiro and Ferreira [15]. This platform allows for the use of algorithms and data structures designed for high performance [14,15], having already been used as a basis for several works [28,29,30,31]. Details related to the developed computational program are described in Appendix C, with an overview of the implemented code being presented in Algorithm A5. This appendix includes the description of a method adopted to favor proper stress redistributions during the cracking process and to avoid any dependence of results on the load increment size, called the most dangerous element method.

**Figure 5:**
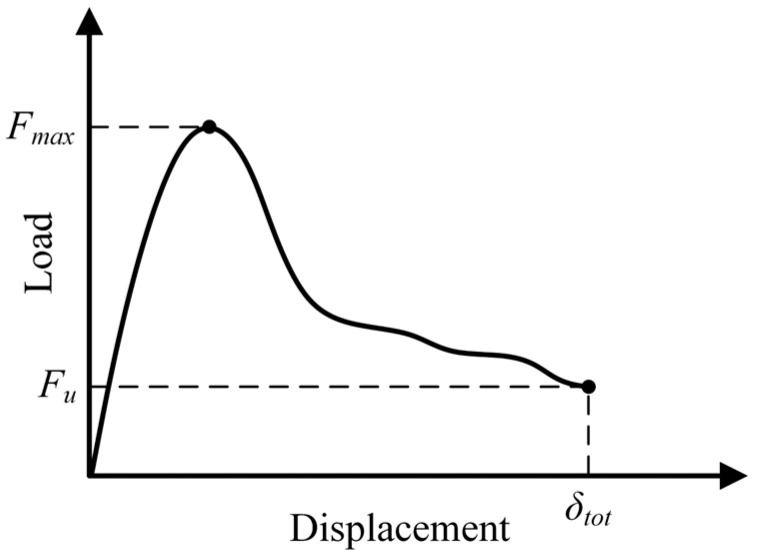
Generic load–displacement diagram, highlighting information that can be used in the simulation stopping criteria: $F_{max}$, $F_{u}$, and $\delta_{tot}$.

The application of the Monte Carlo method requires Algorithm A5 to be executed a number of times, corresponding to a number of Monte Carlo samples whose load–displacement responses may naturally vary considerably from one sample to another. In order to deal automatically with this variation, two simulation stopping criteria were implemented, taking into account some load–displacement diagram information: maximum load ($F_{max}$), ultimate load ($F_{u}$), and ultimate displacement ($\delta_{tot}$), as indicated in Figure 5.

The simulation stopping criteria are named as follows: SSC1, whose evaluation parameter is the ratio between $F_{u}$ and $F_{max}$, with $F_{u}$ being necessarily less than $F_{max}$; and SSC2, whose evaluation parameter is simply $\delta_{tot}$. It is also possible to opt for both criteria simultaneously. More specifically, these criteria are defined as follows:

- SSC1 [$\psi_{a}$] → the criterion is met, and the simulation is terminated, when $\frac{F_{u}}{F_{max}}<\psi_{a}$;
- SSC2 [$\psi_{b}$] → the criterion is met, and the simulation is terminated, when $\delta_{tot}\geq \psi_{b}$.

with the limit parameters $\psi_{a}$ and $\psi_{b}$ being set according to the particularities of the considered analysis, so that $0\leq \psi_{a}<1$ and $\psi_{b}>0$.

It is important to point out that values of $\psi_{a}$ close to 1 should be avoided, since such values do not guarantee the effective knowledge of the peak load. As load fluctuations can naturally occur in the load–displacement diagram, inappropriate values of $\psi_{a}$ can easily cause false peak-load results. For each type of mechanical problem, it is necessary to carry out previous analyses to determine suitable values of $\psi_{a}$.

## 4. Analyzed Cases

**Figure 6:**
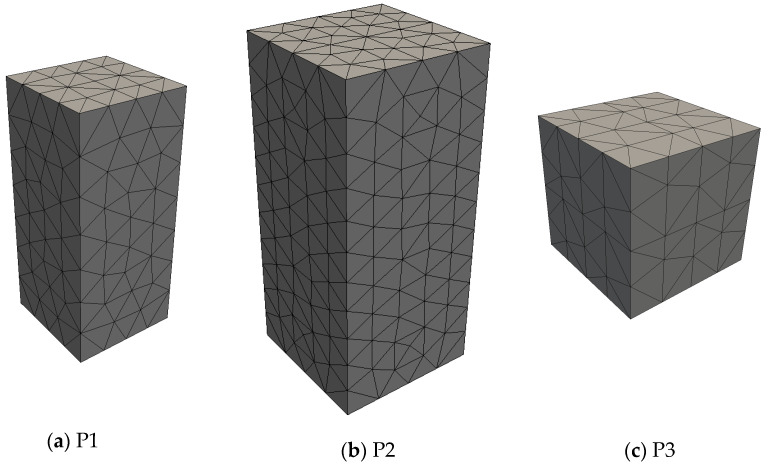
Geometries and meshes for tensile test simulation.

**Figure 7:**
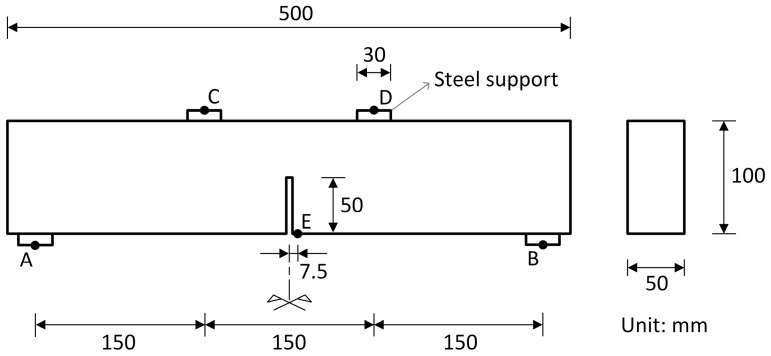
Geometry of the simulated beam (B1).

**Figure 8:**
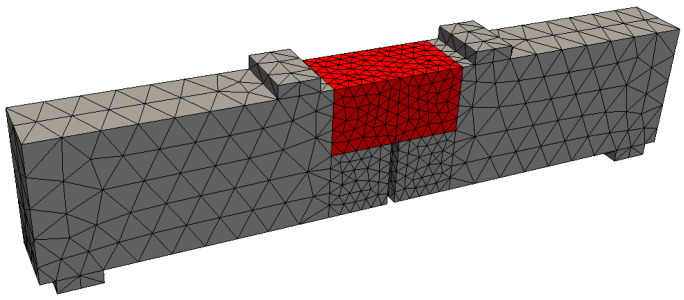
Mesh of the considered beam (B1). Interface elements can only be used in the region highlighted in red, whose length is 100 mm.

The numerical simulations were divided into four stages: (1) mechanical evaluation of the adaptive mesh strategies; (2) inverse analysis; (3) validation; (4) assessment of time savings. Figure 6, Figure 7 and Figure 8 show the geometries and meshes of the analyzed cases, with the details presented in Table 1. In this table, the columns related to interface elements and nodes inform the maximum possible number of these entities in the mesh, remembering that, in case of adaptive mesh use, the informed numbers are not necessarily reached at the end of the analysis; the parameter $2V_{e}$ denotes the average value of the sum of the volumes of the two solid elements that provide the base and the top faces of the interface elements.

In stage 1, direct tensile test simulations were performed by using the square-based prisms (P1, P2, and P3) shown in Figure 6, with P3 corresponding to a cube. Boundary and loading conditions in agreement with the simulated test have been considered, with displacement restrictions being imposed on the base nodes, and displacement increments being applied on the top nodes in the longitudinal direction. Mechanical responses obtained by using the adaptive mesh strategies were compared with the responses provided by the classical strategy of mesh formation. For P1 and P2, the comparisons refer to values of the average, standard deviation, and coefficient of variation of tensile strength, and to the global load–displacement responses. For P3, the comparisons concern the local results of stress redistribution after crack appearance. The purpose of these comparisons was to identify the most suitable adaptive mesh strategy to be adopted in stages 2, 3, and 4, using the classical strategy of mesh formation (CSMF) to provide reference results. The mean tensile strength of each set of Monte Carlo samples ($f_{t}^{mean}$) and the corresponding standard deviation (SD) are calculated by the following expressions [27]:

(9) $$f_{t}^{mean}=\frac{1}{M}\sum_{i=1}^{M}f_{t}^{\left(i\right)}$$

(10) $$SD=\sqrt{\frac{1}{M-1}\sum_{i=1}^{M}{\left(f_{t}^{\left(i\right)}-f_{t}^{mean}\right)}^{2}}$$

in which M is the number of Monte Carlo samples, and $f_{t}^{\left(i\right)}=F_{max}^{\left(i\right)}/A$, where $F_{max}^{\left(i\right)}$ is the maximum load of the i-th Monte Carlo sample, and A is the cross-sectional area.

In stages 2 and 3, the simulations with P1 and P2 were based on the experimental investigation conducted by Rossi et al. [24], which was chosen for the following reasons:

- The study refers to concrete behavior in a direct tensile test, in which the transition from diffuse to localized cracking is the most important phenomenon and the most difficult to capture;
- Concretes with different mechanical properties were produced, enabling the evaluation of different scenarios;
- A large number of experimental samples were tested, implying statistical relevance;
- Experimental results are expressed using Equations (1) and (2) with great accuracy, in such a way that it is completely possible to use these equations to obtain expected values according to the scale effect.

In stage 2, inverse analysis procedures described by Mota et al. [11] were carried out with the purpose of determining the parameters b and c (statistical parameters related to Equations (7) and (8)) for P1 and B1. It is important to highlight that these parameters naturally depend on the volume of the mesh elements, in a manner similar to that in which the values of $f_{t}^{mean}$ and CV given by Equations (1) and (2) depend on the volume of the considered specimens. The parameters b and c are scale effect parameters at the local level, while the values of $f_{t}^{mean}$ and CV provided by Equations (1) and (2) are global-level scale effect results (specimen responses).

**Figure 9:**
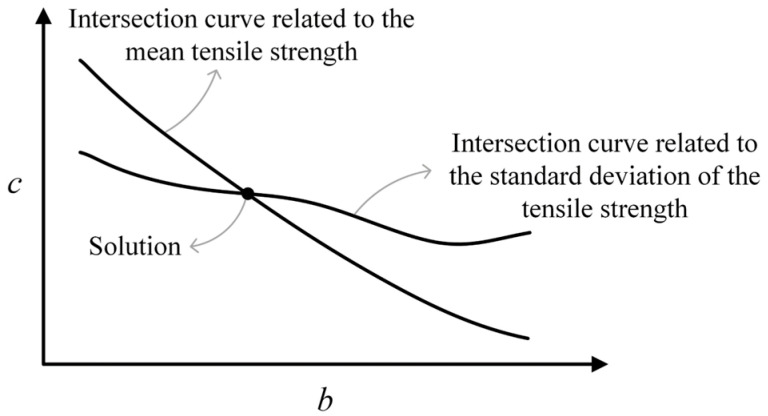
Final step of the inverse analysis procedure for obtaining the most suitable pair of parameters b and c.

Basically, each inverse analysis procedure corresponds to analyzing the same case with different pairs of b and c to define the most appropriate pair of such parameters, taking into account empirical results. A considerable number of Monte Carlo processes is necessary. One Monte Carlo process provides, for example, the mean and the standard deviation of a certain global mechanical response for a given pair of b and c. Repeating this procedure for different pairs of b and c, two surfaces are obtained: one for the mean and another for the standard deviation. These surfaces are properly fitted using polynomial expressions. The expected results for the mean and standard deviation, which can be provided using Equations (1) and (2) for simulations with the prism P1, can be considered as horizontal planes on the b-c domain. In the next step, the intersection curves between these planes and the fitted surfaces must be determined. Figure 9 shows these curves, considering that the tensile strength of the samples, which is given by the maximum load divided by the cross-sectional area, is the mechanical response to be observed. In this figure, the intersection point of the curves represents the most suitable pair of b and c. More details related to the inverse analysis procedures are presented by Mota et al. [11].

For beam B1, detailed in Figure 7 and experimentally tested by Hordijk [32], the boundary and loading conditions were consistent with the simulated four-point bending test, with points A and B indicating the locations of displacement restrictions and points C and D signaling the places of application of displacement increments. Point E marks the deflection measurement location. The acting load was measured as the sum of the forces applied to the supports that contain points C and D. The possibility of using interface elements was restricted to the region highlighted in red in Figure 8. In the simulated test, due to its particularities, the failure is due to tension.

In stage 3, the ability to predict the scale effect was investigated. The values of b and c obtained from an inverse analysis with P1 were used in simulations with P2, which has larger dimensions, for the same concretes. In addition to depending on the material, the parameters b and c, as previously mentioned, depend on the volume of the mesh solid elements. Therefore, the meshes for P1 and P2 were prepared keeping the same average size of elements. In this way, it was possible to evaluate only the effect of increasing the size of the simulated specimen.

Also due to the dependency between the parameters b and c and the size of the elements, the mesh for B1 was generated with the elements of the central region having a very low level of volumetric variation. In relation to P3, there was no need to guarantee low volumetric variation among the elements, since there was no inverse analysis for this case. For this reason, the value of $2V_{e}$ is not important information for P3, as indicated in Table 1.

In stage 4, the efficiency of the adaptive mesh strategy selected in stage 1 was investigated. In order to do this, the simulation times for the analyses performed with the adaptive mesh strategy and the CSMF were compared. As shown in Table 1, the results for P1, P2, and B1 were taken into account in this stage.

Table 2 displays the compressive strength, the modulus of elasticity (E), and the volume of the coarsest aggregate of the considered concretes, as well as the heterogeneity parameters $V_{T}/V_{A}$ and $2V_{e}/V_{A}$. Concretes C1, C2, and C3, experimentally studied by Rossi et al. [24], were applied to P1 and P2. Concrete C4, related to the experimental investigation conducted by Hordijk [32], was applied to B1, for which the value of $2V_{e}/V_{A}$ corresponds to the region in red in Figure 8.

Table 3 contains the values of complementary modeling parameters: the angle of friction, which corresponds to an average estimate based on studies found in the literature [33,34]; the Poisson’s ratio of concretes C1, C2, C3, and C4; and the Poisson’s ratios and moduli of elasticity for P3 and for the steel support of B1. The adopted value for the penalty parameters $k_{11}$, $k_{22}$, and $k_{33}$ related to the constitutive relation of intact interface elements was $1\times 10^{10}\;MN/{dm}^{3}$. This value was chosen from a calibration for which the results of a numerical linear analysis were similar to the analytical ones, without causing problems in the solution.

The nomenclature applied to the Monte Carlo samples involves the geometry, mesh, and concrete in question, according to the terminology used in Table 1 and Table 2. Altogether, there are seven classes of Monte Carlo samples: P1-C1, P1-C2, P1-C3, P2-C1, P2-C2, P2-C3, and B1-C4.

It is worth mentioning that, for the considered probabilistic cracking model, there are some restrictions for performing a mesh sensitivity analysis in which a convergence of results is evaluated as the degree of mesh refinement increases. The variation in the degree of mesh refinement naturally implies a change in the volume of the solid elements. However, the values of b and c depend on the volume of the solid elements (as mentioned before), which means that different values of b and c need to be used when the volume of the solid elements changes, modifying the characteristics of the problem and compromising the mesh sensitivity analysis. A mesh sensitivity analysis can be performed considering the elastic response (in order to ensure the correct elastic response, which is guaranteed in the cases analyzed in this paper) or considering the problem without tensile strength variation (with the same value of tensile strength for all interface elements in the model). In the present paper, the degree of mesh refinement is not a problem, since the adopted meshes provide a level of cracking information that enables the investigation described herein to be carried out. If results with a very high level of cracking information were needed, the meshes could be more refined (which would naturally generate a higher computational cost). But this is not the case in this work, whose purpose is to present a new adaptive mesh strategy and its viability, which does not require results with a very high level of cracking information.

## 5. Results

The results of the analyses conducted in the stages described above are exposed and discussed in the next subsections.

### 5.1. Mechanical Evaluation of the Adaptive Mesh Strategies

For the evaluation of the mesh adaptivity strategies, a total of 80 Monte Carlo samples were simulated for each adaptive mesh strategy as well as for the CSMF. Three pairs of values of b and c that represent different material characteristics were chosen based on previous analyses carried out only to obtain coherent initial values of these parameters: b= 1.2 and c= 12.0 MPa; b= 3.0 and c= 8.0 MPa; and b= 20.0 and c= 7.5 MPa. The following parameters were calculated: mean tensile strength, obtained via Equation (9); standard deviation, determined using Equation (10); coefficient of variation (CV), given by the ratio between SD and $f_{t}^{mean}$; and the discrepancy between $f_{t}^{mean}$ associated with the adaptive mesh strategy in question and $f_{t}^{mean}$ obtained by using the CSMF (${∆}_{1}$). The results for P1 and P2 are presented in Table 4 and Table 5, respectively.

Table 4 and Table 5 show that, for the adaptive mesh strategies, the value of $f_{t}^{mean}$ decreases as the number of interface elements inserted for each new crack increases. In other words, the highest and the lowest value of $f_{t}^{mean}$ are generated with AMS1 and AMS3, respectively. This was observed for the six classes of samples considered in the tables in question. In relation to the comparison with the results of $f_{t}^{mean}$ obtained by using the CSMF, for samples related to P1, Table 4 reveals that the discrepancy ${∆}_{1}$ was between 16.4% and 57.5% for AMS1, between 14.5% and 34.3% for AMS2, and between −0.2% and 1.1% for AMS3. For samples related to P2, Table 5 shows that ${∆}_{1}$ ranged between 21.7% and 58.3% for AMS1, between 19.9% and 37.0% for AMS2, and between −0.1 and 2.2% for AMS3.

The results contained in Table 4 and Table 5 indicate that only AMS3 is capable of generating values of $f_{t}^{mean}$ similar to those produced with the CSMF. Considering the six classes of simulated samples, the maximum absolute value of ${∆}_{1}$ for AMS3, equal to 2.2%, is significantly lower than the value of ${∆}_{1}$ referring to AMS1 and AMS2. Furthermore, the values of SD and CV for AMS3 also have a pronounced similarity with those associated with the CSMF.

It is also important to highlight the occurrence of the scale effect on the values of $f_{t}^{mean}$, SD, and CV for all the considered strategies. In regard to $f_{t}^{mean}$, it can be seen in Table 4 and Table 5 that, for AMS3 for example, the value of this parameter changed from 2.95 MPa in P1-C1 to 2.85 MPa in P2-C1, from 3.77 MPa in P1-C2 to 3.57 MPa in P2-C2, and from 6.13 MPa in P1-C3 to 5.87 MPa in P2-C3. However, scale effect occurrences will be rigorously evaluated in Section 5.3 with values of b and c obtained from inverse analyses, which are necessary for an effective comparison with empirical results.

**Figure 10:**
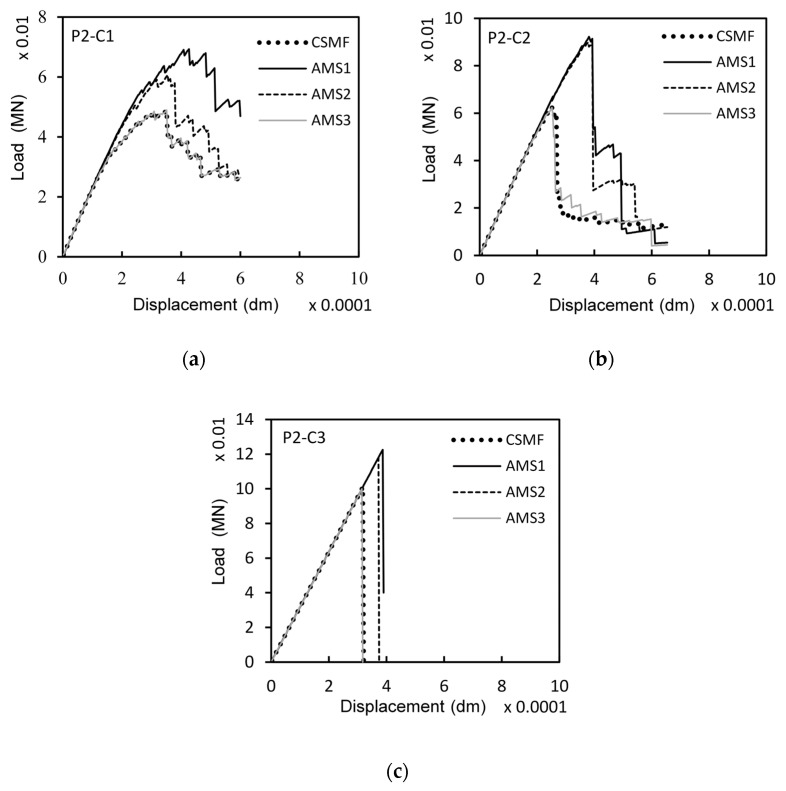
Load–displacement diagrams related to the strategies for interface element usage for a certain Monte Carlo sample of classes (**a**) P2-C1, (**b**) P2-C2, and (**c**) P2-C3.

In order to promote a more specific assessment of the mechanical responses obtained with the adaptive mesh strategies, Figure 10 displays load–displacement diagrams for a certain Monte Carlo sample of classes P2-C1, P2-C2, and P2-C3. It should be mentioned that, for each class, the curves refer to exactly the same sample; that is, the random values of tensile strength at the interfacial regions of solid elements were kept the same for all the strategies.

Figure 10 shows that AMS3 tends to produce load–displacement responses very similar to those generated by the CSMF. As seen in Figure 10b, occasional differences can occur mainly in the post-peak branch. The reason for this is that the stress calculation referring to the interfacial regions of solid elements does not happen in the exact same way for the two strategies (see the process of obtaining the array *Savg* in Appendix B), which may cause different crack propagation paths depending on the random distribution of tensile strength values in the mesh. However, comparing the mechanical responses of other samples, in general, there were no accentuated differences in the post-peak branch, indicating that the CSMF and AMS3 indeed tend to generate similar fracture processes.

Regarding AMS1 and AMS2, Figure 10 demonstrates that both strategies produce load–displacement diagrams markedly different from those generated by the CSMF. Although there is a certain degree of similarity in the shape of the curves, the cracking localization process starts at a higher load level when AMS1 and AMS2 are employed. Consequently, the maximum loads are naturally also higher. This divergent behavior occurs because AMS1 and AMS2 do not generate an amount of intact interface elements in regions close to cracks capable of enabling a stress redistribution process similar to that observed with the CSMF, as discussed next.

#### 5.1.1. Stress Redistribution Verification

**Figure 11:**
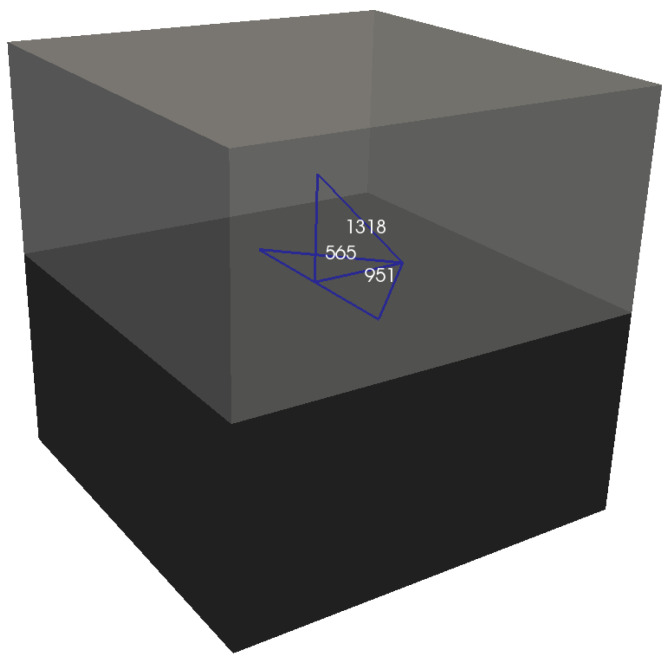
Interfacial regions of solid elements of P3 (highlighted in blue) considered in the stress redistribution verification.

In order to clarify the divergences observed in the mechanical responses obtained with AMS1 and AMS2 in relation to the results provided by the CSMF and AMS3, simulations with P3 were performed taking into account the occurrence of a previously directed crack. The redistribution of stresses in the vicinity of the crack was investigated for each strategy for interface element usage. Figure 11 indicates the three interfacial regions of solid elements considered in such analyses. Regions 565 and 951 are located in the central plane of the cube, and region 1318 is inclined over region 565. Regions 565 and 1318 are located on faces of the same solid element, which corresponds to the top solid element of the interface element associated with region 565.

After imposing a displacement of 6 × 10^−5^ dm on the top face of the cube, a crack was induced in region 565. The normal stress in regions 951 and 1318 was evaluated before and right after the appearance of the crack. The results for each strategy of interface element usage are revealed in Table 6.

It can be seen in Table 6 that the CSMF and AMS3 caused the same level of stress variation in regions 951 (from 1.2 MPa to 1.6 MPa) and 1318 (from 0.8 MPa to 0.5 MPa). This resemblance in the stress redistribution process explains why these strategies tend to produce similar mechanical results, as observed in Table 4 and Table 5 and in Figure 10.

With regard to the analyses carried out with AMS1 and AMS2, Table 6 shows that region 951, without an interface element, did not undergo stress variation, with the stress before and right after the appearance of the crack remaining at a level of 1.2 MPa. On the other hand, in region 1318, which has an interface element, a considerable stress variation was noticed.

In region 951, for AMS1 and AMS2, there was no stress variation, because the change in the strain level of the solid elements in the vicinity of the crack was extremely low. It was observed that the existence of an interface element at an interfacial region close to the crack is necessary so that the stress redistribution is captured with greater sensitivity in this region. Therefore, in the analyses with the CSMF and AMS3, noticeable stress variations, captured by interface elements, were observed in the two examined regions.

#### 5.1.2. Decision on Adaptive Mesh Procedure

It is assumed that the CSMF promotes adequate stress redistribution, since interface elements are previously inserted in all the interfacial regions of solid elements in this strategy, which makes the interface elements capable of capturing post-crack stress redistribution in any part of the mesh. For this reason, an appropriate and coherent adaptive mesh strategy for inserting interface elements efficiently during the analysis must provide results similar to those obtained with the CSMF. According to the evaluation of the adaptive mesh strategies, AMS1 and AMS2 cannot be used to replace the CSMF due to the occurrence of considerable divergences in the stress redistribution processes, resulting in important differences in the mechanical responses. Nevertheless, these results are of great relevance, since they can prevent these strategies from being used by other researchers in future investigations. On the other hand, the results generated with AMS3 show a high degree of equivalence with those obtained through the CSMF, which is justified by the similarity in the redistribution of stresses. Thus, considering the need to submit the mesh adaptivity to other assessments, AMS3 was chosen as the most suitable adaptive mesh strategy to continue the investigation.

AMS3 is markedly different from other adaptive mesh strategies involving interface elements found in the literature [16,17,18,19,20,21]. Such a difference is mainly due to the fact that these works do not take into account the influence of the adaptive mesh strategy on the redistribution of stresses after cracking. In these works, there is no comparison between the results generated with the proposed adaptive mesh strategy and the results obtained with interface elements in all the interfacial regions of solid elements from the beginning of the analysis.

It is important to highlight that, as the probabilistic nature of the cracking model used here did not affect the development of the adaptivity strategies, AMS3 can also be applied to purely deterministic cracking models that employ interface elements.

### 5.2. Inverse Analysis

The values of b and c obtained through inverse analysis procedures for samples related to P1 and B1 are shown in Table 7. For the samples related to P1, values of $f_{t}^{mean}$ and CV given by Equations (1) and (2) were taken into account. For B1-C4, the inverse analysis was based on values of the maximum load and work of fracture extracted from an experimental curve. It can be noticed that the values of b and c determined with AMS3 are very similar to those obtained using the CSMF, with an absolute discrepancy of less than 2%. Naturally, the statistical parameters are not exactly the same for the two strategies due to the possibility of small differences in the mechanical responses, as previously discussed.

In order to verify the accuracy of the performed inverse analyses, the values of b and c presented in Table 7 were used to simulate 80 Monte Carlo samples for each of classes P1-C1, P1-C2, and P1-C3 and 20 Monte Carlo samples of class B1-C4. The results are displayed in Table 8 and Table 9. The variable ${∆}_{2}$ in Table 8 denotes the discrepancy between the numerical modeling result referring to $f_{t}^{mean}$, obtained through Equation (9), and the empirical value of $f_{t}^{mean}$, determined using Equation (1). In Table 9, $F_{max}^{exp}$ and $W_{f}^{exp}$ represent, respectively, the experimental values of the maximum load and work of fracture until the deflection of 0.3 mm, $F_{max}^{mean}$ and $W_{f}^{mean}$ denote the numerical modeling results for the mean maximum load and mean work of fracture until the mentioned deflection respectively, and ${∆}_{3}$ refers to the discrepancy between $F_{max}^{mean}$ and $F_{max}^{exp}$. It can be seen in both tables that all the numerical modeling results closely resemble the empirical or experimental data. The absolute values of ${∆}_{2}$ and ${∆}_{3}$ are less than 2%, associating the inverse analyses with a high degree of accuracy.

**Figure 12:**
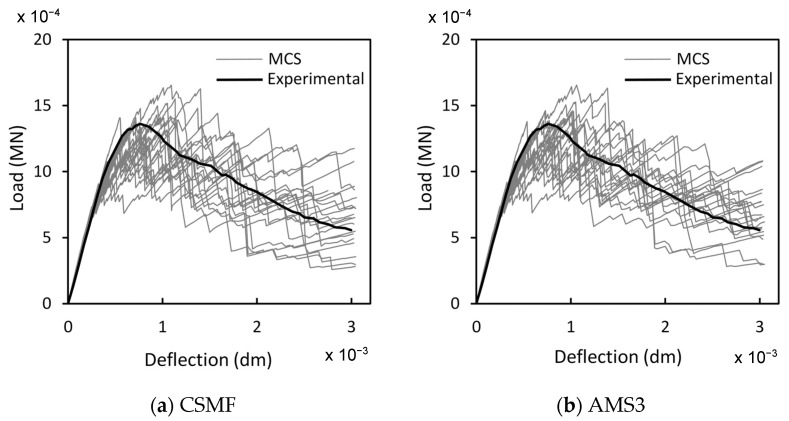
Load–deflection diagrams of 20 samples B1-C4 for simulations with (**a**) the CSMF and (**b**) AMS3.

Figure 12 shows the load–deflection diagrams of the 20 Monte Carlo samples (MCS) of class B1-C4, considered in Table 9, for simulations with the CSMF and AMS3. The experimental response is also displayed. As expected, although the values of b and c for B1-C4 were obtained with AMS3, these values can be normally used in simulations with the CSMF, with the mechanical responses for both strategies being quite similar, mainly in terms of the average results, as indicated in Table 9.

### 5.3. Validation

The validation analyses consisted in simulating 80 Monte Carlo samples for each of the classes P2-C1, P2-C2, and P2-C3, with the values of b and c presented in Table 7. The results are revealed in Table 10, with ${∆}_{2}$ being the discrepancy between numerical modeling and empirical values for $f_{t}^{mean}$, as previously defined. Again, a strong similarity is observed between the results provided by the CSMF and AMS3. By comparing the numerical modeling results shown in Table 10 with those displayed in Table 8, it is possible to notice the scale effect on the values of $f_{t}^{mean}$, SD, and CV for the three analyzed classes, which was already expected, taking into account the results discussed in Section 5.1.

In Table 10, the values of ${∆}_{2}$ range from −4.3% to 8.6%, which can be judged as a good result, considering the high complexity of the probabilistic scale effect linked to the value of $f_{t}^{mean}$. The numerical modeling results for SD and CV also show good similarity with the empirical ones. Thus, it can be seen that the adaptive probabilistic model, as well as the classical alternative, is capable of predicting the scale effect at a level similar to that experimentally observed.

**Figure 13:**
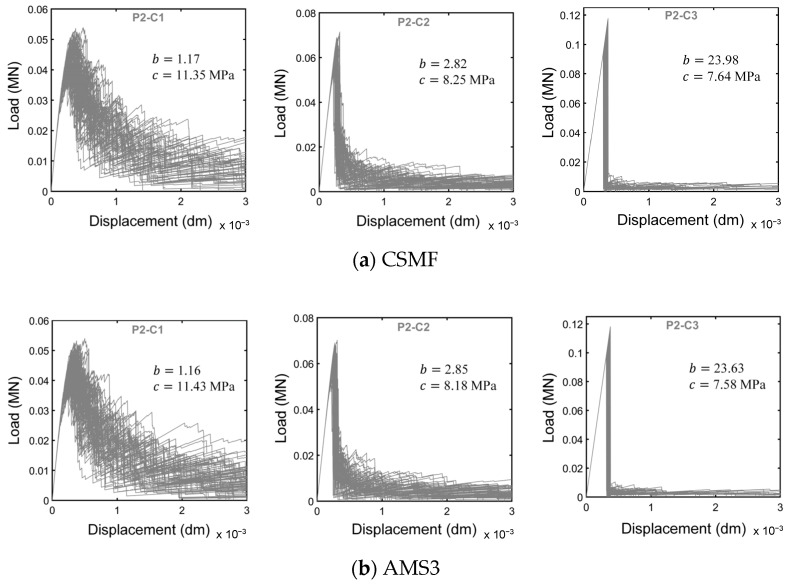
Load–displacement diagrams of 80 Monte Carlo samples of classes P2-C1, P2-C2, and P2-C3 for simulations with (**a**) the CSMF and (**b**) AMS3.

**Figure 14:**
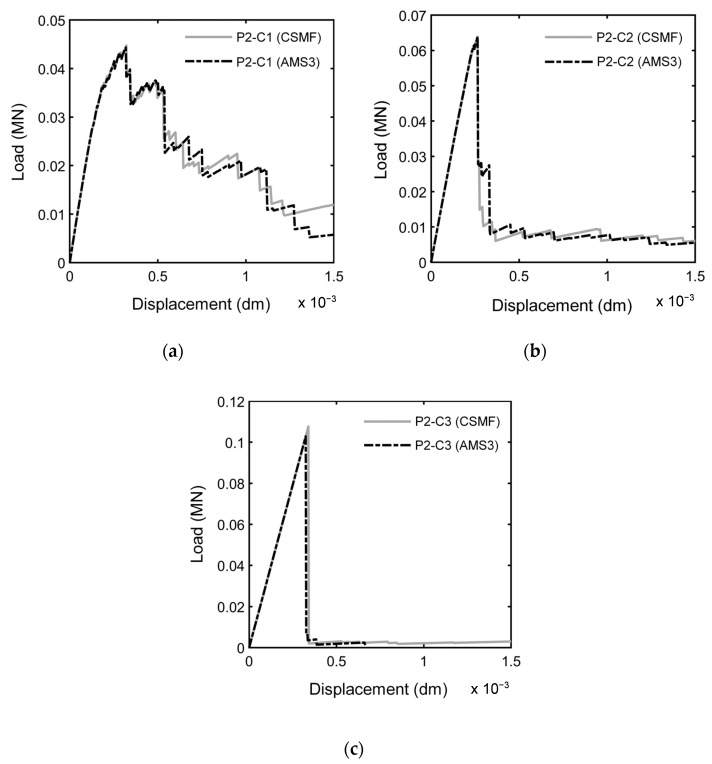
Comparison between typical load–displacement diagrams obtained by using the CSMF and AMS3 for samples (**a**) P2-C1, (**b**) P2-C2, and (**c**) P2-C3.

For a general assessment of the mechanical responses produced with the CSMF and AMS3, Figure 13 presents the 80 load–displacement diagrams obtained for each class of Monte Carlo samples indicated in Table 10 for both strategies. With the purpose of enabling detailed examinations, Figure 14 displays the typical curves for each class until the displacement of 1.5 × 10^−3^ dm. In this paper, a typical curve is one of the curves of the set of Monte Carlo sample curves that has characteristics that makes it close to an average curve. In the mentioned figures, the resemblance between the responses produced with the CSMF and AMS3 is clear, and occasional divergences between individual behaviors are easily compensated by the application of the Monte Carlo method. It is important to observe that parameter b exerts a strong influence on the results. Low values of this parameter are associated with high variability in the obtained diagrams. Moreover, the higher the value of b, the lower the level of nonlinearity in the pre-peak response and the lower the prominence of softening behavior. It can still be noted that the results reflect the very well-known phenomenon of brittleness growth under uniaxial tension with the increase in compressive strength.

**Figure 15:**
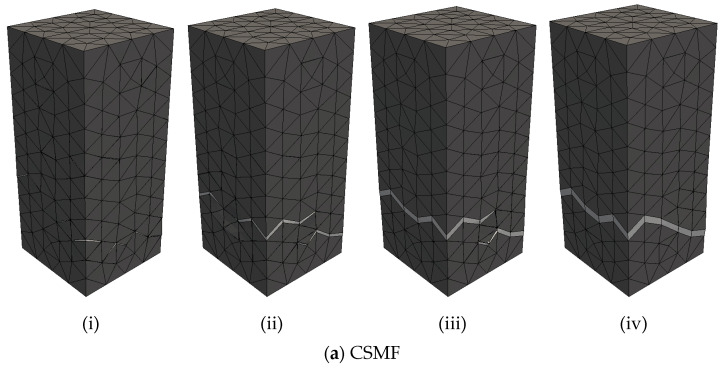

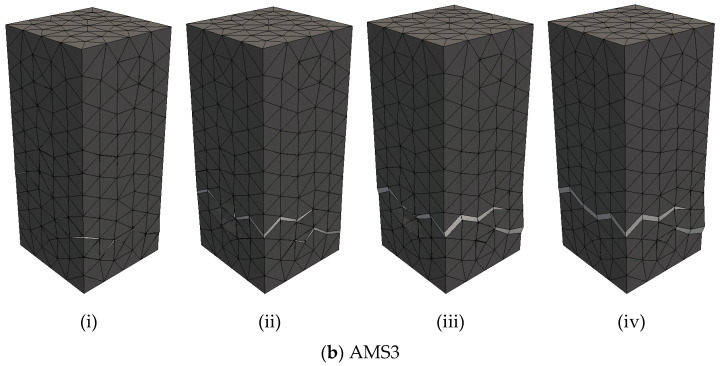
Crack patterns of sample P2-C2 obtained by using (**a**) the CSMF and (**b**) AMS3 for (**i**) the peak load and (**ii**,**iii**,**iv**) post-peak loads.

As expected, the similarity in the load–displacement diagrams produced with the CSMF and AMS3 corresponds to a similarity in the crack patterns generated by both strategies, as illustrated in Figure 15, for a P2-C2 sample. In this figure, the cracking process is depicted by means of crack patterns corresponding to peak and post-peak loads. The localized fracture process, which involves cracking localization and crack propagation, is clearly noticed. As can be seen, the main crack did not develop at half the height of the specimen, which is a natural consequence of the random assignment of tensile strength values. If the rupture region is not previously directed, the main crack can appear anywhere along the height of the specimen, such as what occurs in experimental tests. A natural characteristic of the explicit cracking modeling approach is that the geometrical representation of cracks depends on the degree of refinement of the mesh. Therefore, the more refined the mesh, the more cracking information can be obtained. The degree of mesh refinement adopted in the present work is in accordance with the level of cracking information required for the investigation.

### 5.4. Assessment of Time Savings

The efficiency of AMS3, in terms of computational execution time, was evaluated by means of discrepancy between the average simulation times related to this strategy and to the CSMF. This discrepancy is denoted by ${∆}_{4}$. Negative values of ${∆}_{4}$ refer to time savings, and, naturally, the higher the modulus of these values, the higher the efficiency of AMS3.

A computer with the basic characteristics reported in Table 11 was used to measure the simulation times. Table 12 displays the simulated classes, together with basic information about the performed analyses: the parameters b and c, whose values, for samples related to P1 and P2, are the same as those adopted in Section 5.1; the number of Monte Carlo samples (M); and the simulation stopping criterion (SSC) employed for each class, exposed according to definitions expressed in Section 3.3.

Table 13 reveals the results referring to the efficiency of AMS3, which are the following: the average time required to simulate a Monte Carlo sample of the considered class, for the CSMF and AMS3; the discrepancy ${∆}_{4}$; and the parameter η, defined as the average percentage ratio between the number of interface elements present in the mesh at the end of the simulation with AMS3 and the maximum possible number of these elements in the analyzed case.

In Table 13, the negative values of ${∆}_{4}$ indicate an efficiency of AMS3 for all the classes. The smallest moduli of ${∆}_{4}$, 3.2% and 8.6%, are associated with very high values of η, while the largest moduli, 72.9% and 76.9%, are related to low values of η. There is coherence in this relationship between ${∆}_{4}$ and η, since high values of η denote that the amount of interface elements used in AMS3 become similar to that used in the CSMF, with AMS3 having time-consumption conditions similar to those observed in the CSMF. In this way, when the number of interface elements in AMS3 quickly approaches the maximum possible value, which happens with P1-C1 and P2-C1 (but mainly with P1-C1), this strategy shows low efficiency, tending to cause relatively small reductions in the simulation times. On the other hand, for values of η smaller than 85%, the average time savings are very significant. Thus, AMS3 proved to be noticeably advantageous, demonstrating an efficiency that naturally depends on the characteristics of the analyzed problem.

**Figure 16:**
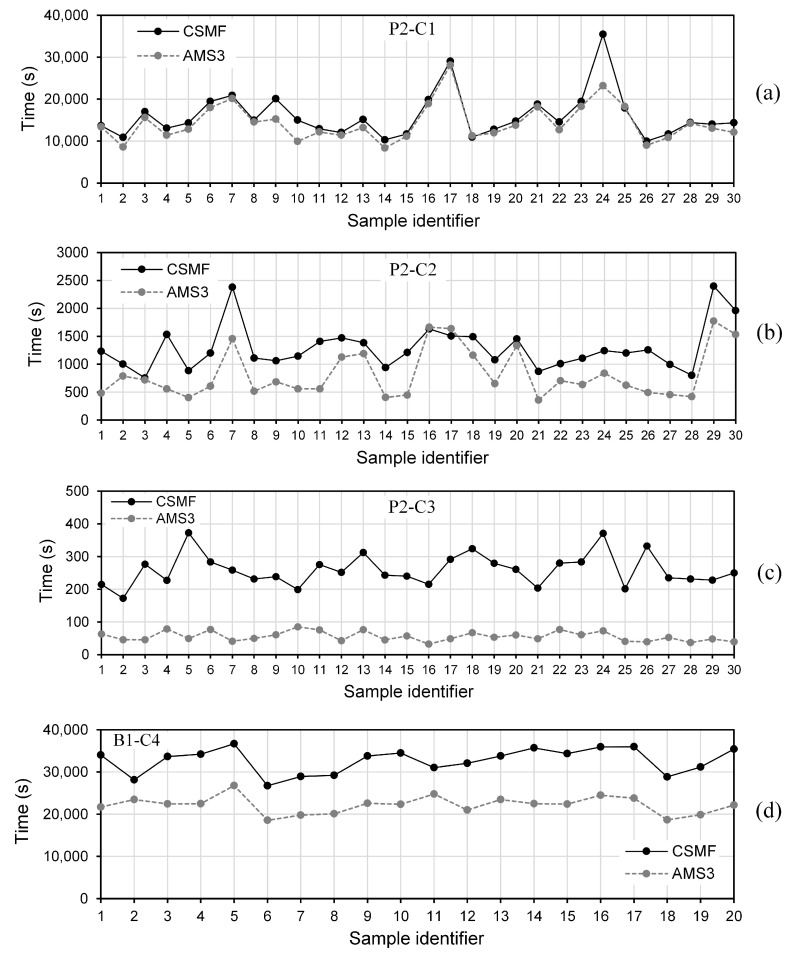
Individual simulation times of samples (**a**) P2-C1, (**b**) P2-C2, (**c**) P2-C3, and (**d**) B1-C4 for the CSMF and AMS3.

Individual results can be examined in Figure 16, which reveals the individual simulation times of 30 samples for P2-C1, P2-C2, and P2-C3, as well as the simulation times of the 20 B1-C4 samples considered in the analyses, for the CSMF and AMS3. In this figure, the difference in the simulation times for the same class of Monte Carlo samples is a natural consequence of the adopted probabilistic approach. Depending on the distribution of random tensile strength values, the crack pattern of the Monte Carlo samples can be very different, leading to distinct load–displacement diagrams (see Figure 13), similar to what happens in real experiments. Different load–displacement diagrams can be related to very different numbers of nonlinear iterations and, consequently, very different simulation times.

As can be seen in Figure 16b for samples 16 and 17, it is possible that, very infrequently, the simulation time for AMS3 is longer than that for the CSMF. The explanation for this is that, as previously justified, the stress calculation referring to the interfacial regions of solid elements does not happen in the exact same way in these strategies, so occasional differences between the mechanical behaviors produced by them can occur, mainly in the post-peak branch (see Figure 10b). Thus, the crack propagation process may not be exactly the same for both strategies, directly influencing the simulation times associated with them. For the same reason, the time reductions generated by AMS3 for some samples were considerably greater than the average reductions indicated in Table 13, as noticed, for example, in Figure 16a for sample 24 and in Figure 16b for sample 4. These observations reinforce the importance of evaluating the efficiency of AMS3 through average simulation times, favoring the statistical compensation of infrequent results.

In classes where practically the entire fracture process is concentrated in a delimited region of the mesh (P1-C3, P2-C3, and B1-C4), AMS3 saved a marked amount of time for absolutely all the analyzed samples, in accordance with what can be seen in Figure 16c for samples P2-C3 and in Figure 16d for samples B1-C4. It is important to note that the concentrated fracture process had different causes: predominantly sudden failure, for samples P2-C3, for example, as illustrated in Figure 10c; and presence of a notch, for samples B1-C4.

## 6. Conclusions

This paper communicates the development of an adaptive mesh strategy to optimize the use of interface elements in a 3D probabilistic explicit cracking model for concrete. The investigation involved the evaluation of three adaptive mesh strategies: AMS1, AMS2, and AMS3. Based on comparisons between mechanical behaviors, only AMS3 proved to be able to replace the classical strategy of mesh formation (CSMF) generally adopted in probabilistic explicit cracking models.

AMS1 and AMS2 are not suitable to replace the CSMF due to divergences in the redistribution of stresses during fracturing, which lead to remarkably different mechanical responses. On the other hand, the mechanical responses produced by using AMS3 tend to present a very high degree of equivalence with those obtained through the CSMF, as there is similarity in the redistribution of stresses. Thus, an important conclusion is that different adaptive mesh strategies can lead to distinct stress redistribution processes and, consequently, to very different mechanical responses.

Possible differences between the mechanical responses related to AMS3 and the CSMF are basically concentrated in the post-peak behavior. Such differences are generally of little importance and stem from the fact that the calculation of stresses in interfacial regions of solid elements does not happen in the exact same way for the two strategies. Nevertheless, with the application of the Monte Carlo method, the divergent behaviors tend to compensate for each other, so that there is convergence on very similar average results.

The adaptive probabilistic model based on AMS3, as well as the classical alternative, is capable of predicting the scale effect at a level similar to that experimentally observed, besides covering the occurrence of different levels of softening behavior.

Regarding the efficiency of AMS3, in terms of the computational execution time, it can be concluded that this adaptive procedure is advantageous. This strategy reduced the average simulation time for all the analyzed cases. Understandably, the level of efficiency depends on the characteristics of the considered problem. The smallest average time savings, in percentage terms, were observed for cases in which the number of interface elements quickly reached values very close to the maximum possible number. In cases where this did not occur, there were significant time savings, corresponding to reductions in the average simulation time that ranged from 32% to 77%.

It is also important to highlight that, as the probabilistic nature of the cracking model used here did not affect the development of the adaptivity strategies, AMS3 can also be applied to purely deterministic cracking models that employ interface elements.

## Figures and Tables

**Table 1:** Details relating to geometries and meshes of the considered computational samples.

Geometry	Stages *	Length (dm)	Cross-Section (dm^2^)	Solid Elements	Interface Elements	Nodes	$2V_{e}$ (10^−3^ dm^3^)
P1	1, 2, 4	2.2	0.9503	837	1522	8370	5.0
P2	1, 3, 4	3.0	1.7671	2141	4010	21,410	5.0
P3	1	1.0	1.0	527	958	5270	---
B1	2, 4	5.0	---	8496	6071	39,716	0.16

* Stages: 1—mechanical evaluation of the adaptive mesh strategies; 2—inverse analysis; 3—validation; 4—assessment of time savings.

**Table 2:** Mechanical properties and heterogeneity parameters of the simulated concretes.

Concretes	$f_{c}$ (MPa)	E (GPa)	$V_{A}$ (10^−3^ dm^3^)	$V_{T}/V_{A}$		${2V}_{e}/V_{A}$	
				P1	P2	P1 and P2	B1
C1	35.0	39.8	4.2	499.1	1265.6	1.2	---
C2	55.8	45.3					
C3	127.5	53.9					
C4	48.1	38.0	0.27	---	---	---	0.6

**Table 3:** Complementary modeling parameters.

Parameters	Value
Angle of friction	36°
Poisson’s ratio of concretes	0.2
Poisson’s ratio for simulation of P3	0.2
Modulus of elasticity for simulation of P3	20 GPa
Poisson’s ratio for the steel support of B1	0.3
Modulus of elasticity for the support of B1	200 GPa

**Table 4:** Results of the mechanical evaluation of the adaptive mesh strategies for samples related to P1.

Samples	b	c (MPa)	Strategy	$f_{t}^{mean}$ (MPa)	SD (MPa)	CV	${∆}_{1}$ (%)
P1-C1	1.2	12.0	CSMF	2.96	0.30	0.103	---
			AMS1	4.66	0.43	0.092	57.5
			AMS2	3.86	0.39	0.102	30.7
			AMS3	2.95	0.30	0.102	−0.2
P1-C2	3.0	8.0	CSMF	3.75	0.24	0.063	---
			AMS1	5.45	0.34	0.063	45.2
			AMS2	5.04	0.33	0.065	34.3
			AMS3	3.77	0.24	0.063	0.6
P1-C3	20.0	7.5	CSMF	6.07	0.33	0.054	---
			AMS1	7.06	0.16	0.023	16.4
			AMS2	6.95	0.17	0.025	14.5
			AMS3	6.13	0.35	0.057	1.1

**Table 5:** Results of the mechanical evaluation of the adaptive mesh strategies for samples related to P2.

Samples	b	c (MPa)	Strategy	$f_{t}^{mean}$ (MPa)	SD (MPa)	CV	${∆}_{1}$ (%)
P2-C1	1.2	12.0	CSMF	2.85	0.20	0.070	---
			AMS1	4.51	0.33	0.074	58.3
			AMS2	3.80	0.29	0.078	33.3
			AMS3	2.85	0.20	0.069	−0.1
P2-C2	3.0	8.0	CSMF	3.56	0.21	0.058	---
			AMS1	5.29	0.27	0.050	48.8
			AMS2	4.87	0.27	0.054	37.0
			AMS3	3.57	0.20	0.057	0.4
P2-C3	20.0	7.5	CSMF	5.75	0.26	0.045	---
			AMS1	7.00	0.15	0.021	21.7
			AMS2	6.89	0.16	0.023	19.9
			AMS3	5.87	0.27	0.046	2.2

**Table 6:** Stress redistribution results for a crack in region 565 of P3.

Strategy	Stress in Region 951 (MPa)		Stress in Region 1318 (MPa)	
	Before the Crack	After the Crack	Before the Crack	After the Crack
CSMF	1.2	1.6	0.8	0.5
AMS1	1.2	1.2	0.8	0.3
AMS2	1.2	1.2	0.8	0.3
AMS3	1.2	1.6	0.8	0.5

**Table 7:** Weibull distribution parameters b and c obtained via inverse analysis.

Samples	Strategy	b	c (MPa)
P1-C1	CSMF	1.17	11.35
	AMS3	1.16	11.43
P1-C2	CSMF	2.82	8.25
	AMS3	2.85	8.18
P1-C3	CSMF	23.98	7.64
	AMS3	23.63	7.58
B1-C4	AMS3	1.01	12.42

**Table 8:** Verification of the accuracy of the inverse analyses carried out with samples P1-C1, P1-C2, and P1-C3.

Samples	Empirical			Strategy	Numerical			${∆}_{2}$ (%)
	$f_{t}^{mean}$ (MPa)	SD (MPa)	CV		$f_{t}^{mean}$ (MPa)	SD (MPa)	CV	
P1-C1	2.72	0.31	0.11	CSMF	2.73	0.29	0.11	0.05
				AMS3	2.72	0.28	0.10	−0.1
P1-C2	3.73	0.29	0.08	CSMF	3.77	0.29	0.08	1.2
				AMS3	3.77	0.31	0.08	1.3
P1-C3	6.41	0.30	0.05	CSMF	6.45	0.28	0.04	0.6
				AMS3	6.45	0.28	0.04	0.6

**Table 9:** Verification of the accuracy of the inverse analyses carried out with samples B1-C4.

Samples	Experimental		Strategy	Numerical		${∆}_{3}$ (%)
	$F_{max}^{exp}$ (kN)	$W_{f}^{exp}$ (kN∙mm)		$F_{max}^{mean}$ (kN)	$W_{f}^{mean}$ (kN∙mm)	
B1-C4	1.36	0.26	CSMF	1.36	0.26	−0.17
			AMS3	1.36	0.26	−0.12

**Table 10:** Validation results for samples P2-C1, P2-C2, and P2-C3.

Samples	Empirical			Strategy	Numerical			${∆}_{2}$ (%)
	$f_{t}^{mean}$ (MPa)	SD (MPa)	CV		$f_{t}^{mean}$ (MPa)	SD (MPa)	CV	
P2-C1	2.39	0.23	0.10	CSMF	2.60	0.22	0.09	8.6
				AMS3	2.59	0.22	0.09	8.4
P2-C2	3.43	0.22	0.06	CSMF	3.56	0.20	0.06	4.0
				AMS3	3.53	0.21	0.06	3.1
P2-C3	6.39	0.22	0.03	CSMF	6.12	0.24	0.04	−4.3
				AMS3	6.21	0.25	0.04	−2.8

**Table 11:** Basic characteristics of the computer used to measure simulation times.

Processor	Intel^®^ Xeon^®^ E5-2630 v4 (2 units)
Total cores	20 (10 per processor)
Frequency	2.2 GHz (fixed in the analyses)
RAM	16 GB DDR4-2133 MHz (16 units, totaling 256 GB)
Motherboard	Intel^®^ S2600CWR
Operational system	CentOS Linux 7

**Table 12:** Basic information about the analyses performed to evaluate the efficiency of AMS3.

Samples	b	c (MPa)	M	SSC
P1-C1	1.2	12.0	80	SSC1 [0.40]
P1-C2	3.0	8.0	80	
P1-C3	20.0	7.5	80	
P2-C1	1.2	12.0	80	SSC1 [0.50]
P2-C2	3.0	8.0	80	
P2-C3	20.0	7.5	80	
B1-C4	1.01	12.42	20	SSC2 [0.3 mm]

**Table 13:** Results referring to the efficiency of AMS3.

Samples	Average Time (s)		${∆}_{4}$ (%)	η (%)
	CSMF	AMS3		
P1-C1	2589	2507	−3.2	99.5
P1-C2	196	132	−32.8	81.5
P1-C3	38	10	−72.9	37.7
P2-C1	16,032	14,652	−8.6	99.7
P2-C2	1387	856	−38.3	83.2
P2-C3	254	58	−76.9	28.1
B1-C4	32,707	22,168	−32.2	82.0

## Data Availability

The data related to this this study are available in the article.

## Appendix A. Algorithm for Generation and Distribution of Random Tensile Strength

The random values of tensile strength are generated and distributed to interfacial regions of solid elements according to Algorithm A1, with storage occurring in the array *Str*.

**Algorithm A1.** Basic steps for generation and distribution of random tensile strength
1. *Set the values of b and c;* 2. n=0; *// interfacial region identifier* 3. **do** (*until the last interfacial region of solid elements*) 4. n=n+1; 5. *Generate a random number y between 0 and 1 from uniform distribution*; 6. *Compute the random number x using Equation (8)*; 7. *Str*(n) =x; *//* tensile strength of the n*-th interfacial region* 8. **end**

## Appendix B. Computational Implementation of the Adaptive Mesh Strategies

Regarding the computational implementation of the adaptive mesh strategies, two arrays determined in the pre-processing phase can be highlighted initially: *Rmap* and *Ad*. The array *Rmap* maps all interfacial regions of solid elements, correlating the interfacial regions with the faces of the solid elements. The array *Ad* gathers all the solid elements adjacent to each interfacial region. The determination of these arrays is based on the idea that each interfacial region is associated with a possible interface element in the mesh.

In *Rmap*, interfacial regions under global numbering relate to faces of solid elements under local numbering. Generically, the term *Rmap*(*t*, *q*) corresponds to the identifier number of the interfacial region located on the *t*-th face of the solid element *q*, with *Rmap*(*t*, *q*) = 0 indicating that there is no interfacial region on the face in question.

The array *Ad* is also associated with the global numbering of the interfacial regions, so that the term *Ad*(*n*, *j*) designates the *n*-th solid element adjacent to the interfacial region *j*. Specifically, the term *Ad*(1, *j*) represents the solid element that provides the base face, and *Ad*(2, *j*) is the one that provides the top face of the interface element that may be inserted in the interfacial region *j*. The other neighboring solid elements are stored in positions given by *n* > 2.

In the processing phase, other arrays are of great importance, namely *Ieac*, which signals the interfacial regions with interface elements, as well as their cracking state; *Savg*, which stores the average normal stresses in interfacial regions, taking into account that positive values correspond to tensile stresses; and *Intc*, which indicates the detection of new cracks in interfacial regions without interface elements or with intact interface elements.

In order to facilitate recurring queries, the generic terms of the arrays mentioned above are described in Table A1.

**Table A1:** Generic terms of arrays used in the computational implementation of the adaptive mesh strategies.

Generic Terms	Description
*Rmap*(*t*, *q*)	Identifier number of the interfacial region located on the *t*-th face of the solid element *q*.
*Ad*(*n*, *j*)	*n*-th solid element adjacent to the interfacial region *j*.
*Str*(*j*)	Tensile strength of the interfacial region *j*.
*Ieac*(*j*)	Code for indicating the presence of interface element at the interfacial region *j*; *Ieac*(*j*) = 0 indicates that there is no interface element, *Ieac*(*j*) = 1 signals the presence of intact interface element, and *Ieac*(*j*) = 2 informs the existence of cracked interface element in region *j*.
*Savg*(*j*)	Average normal stress acting in the interfacial region *j*; this term is calculated for *Ieac*(*j*) = 0 or *Ieac*(*j*) = 1.
*Intc*(*j*)	Code for indicating the detection of new crack at the interfacial region *j*. *Intc*(*j*) = 0 indicates that no new crack was detected at the interfacial region *j* or that there is already a cracked interface element at this region, and *Intc*(*j*) = 1 signals the detection of a new crack at the region in question; the array *Intc* is updated at each iteration of the nonlinear solution procedure.

The array *Savg* is determined for interfacial regions without interface elements or with intact interface elements. For an interfacial region without an interface element, firstly, the nodal stresses are calculated. Then, an average stress tensor for this region is obtained from the contributions of all the nodes contained in it, with the stresses being added and averaged. Subsequently, this tensor is rotated according to the vector normal to the interfacial region plane. For an interfacial region with an intact interface element, the average normal stress is calculated directly at the interface element, taking into account its constitutive relation.

Algorithm A2 summarizes the determination of *Intc* at each nonlinear solution iteration. As informed in line 1, the input parameters are the arrays *Ieac* and *Str*. The array *Savg* is determined in line 3 according to steps described above. In this line, only the stresses for interfacial regions without interface elements are calculated, because the stresses for interfacial regions with intact interface elements are previously calculated. The detection of new cracks takes place in line 11 by considering the tensile strength ($f_{t}^{int}$) and the acting normal stress ($\sigma_{int}$) at the interfacial region in question. It should be noted that line 7 guarantees that, at the end of the algorithm execution, the code stored in *Intc* can be equal to 1 only for interfacial regions without cracked interface elements.

**Algorithm A2.** Determination of *Intc*
1. Input: *Ieac* and *Str* 2. Output: *Intc* 3. *Determine the array Savg*; 4. j= 0; *// interfacial region identifier* 5. **do** (*until the last interfacial region*) 6. j= *j +* 1; 7. *Intc*(*j*) =0; 8. **if** *Ieac*(*j*) = 0 or *Ieac*(*j*) = 1 **then** 9. $f_{t}^{int}=$ *Str*(*j*); 10. $\sigma_{int}=$ *Savg*(*j*); *// normal stress in the interfacial region j* 11. **if** $\sigma_{int}\geq f_{t}^{int}$ **then** *Intc*(*j*) = 1; 12. **end** 13. **end**

Algorithm A3 describes the basic steps for performing each adaptive mesh strategy (AMS) at each iteration of the nonlinear solution procedure. The input information, quoted in line 1, is the array *Intc* defined by Algorithm A2, the array *Ieac* (outdated), and the arrays *Ad* and *Rmap*. The output parameter is the array *Ieac* (updated), as indicated in line 2. The array *Intc* is used in line 6 for the purpose of selecting the interfacial regions in which new cracks were detected, with the insertion of cracked interface elements occurring in line 7. The array *Ad* is used in lines 10, 14, and 21, indicating the solid elements that will have their faces considered for the introduction of intact interface elements according to the particularities of each adaptive mesh strategy. In lines 11, 15, and 22, Algorithm A4 is used for inserting intact interface elements at interfacial regions adjacent to the cracked interface element.

**Algorithm A3.** Basic steps of the adaptive mesh strategies
1. Input: *Intc, Ieac, Ad* and *Rmap* 2. Output: *Ieac* 3. j= 0; *// interfacial region identifier* 4. **do** (*until the last interfacial region*) 5. j= *j +* 1; 6. **if** *Intc*(*j*) = 1 **then** 7. *Insert a cracked interface element at the interfacial region j*; 8. *Ieac*(*j*) = 2; 9. **if** AMS = AMS1 **then** 10. q= *Ad*(*2, j*); *// top solid element* 11. *Utilize Algorithm A4, providing q, Rmap and Ieac*; 12. **else if** AMS = AMS2 **then** 13. **do** (for *n* = 1 and *n* = 2) 14. q= *Ad*(*n, j*); 15. *Utilize Algorithm A4, providing q, Rmap and Ieac*; 16. **end** 17. **else if** AMS = AMS3 **then** 18. *n* = 0; *// identifier of the solid element adjacent to the region j* 19. **do** (*until the last solid element adjacent to the interfacial region j*) 20. n= *n +* 1; 21. q= *Ad*(*n, j*); 22. *Utilize Algorithm A4, providing q, Rmap and Ieac*; 23. **end** 24. **end** 25. **end** 26. **end**

**Algorithm A4.** Insertion of intact interface elements at interfacial regions located on faces of the solid element *q*
1. Input: *q*, *Rmap* and *Ieac* 2. Output: *Ieac* 3. **do** (for *t* = 1, …, 4); *// loop on the element faces* 4. m= *Rmap*(*t*, *q*); *// interfacial region corresponding to face t* 5. **if** m> 0 and *Ieac*(*m*) = 0 **then** 6. *Insert an intact interface element at the interfacial region m*; 7. *Ieac*(*m*) = 1; 8. **end** 9. **end**

In Algorithm A4, in line 4, the array *Rmap* indicates the solid element faces on which intact interface elements should be placed, with line 5 ensuring that only faces corresponding to interfacial regions without interface elements are taken into account. After that, the insertion of intact interface elements happens in line 6. It is important to note that, even when the crack is detected at an interfacial region containing an intact interface element, there is the possibility of inserting new intact interface elements around the crack. Thus, the pattern of disposition of interface elements around the crack in each adaptivity strategy is preserved.

The array *Ieac* is updated in line 8 of Algorithm A3 and in line 7 of Algorithm A4 according to the codification described in Table A1. These updates are of fundamental relevance not only to Algorithms A3 and A4 but also to Algorithm A2, which also has the array *Ieac* as input information.

## Appendix C. Computational Program Developed for the Analyses

Algorithm A5 provides an overview of the developed computational program. The first step is to read the analysis configuration information: strategy for interface element usage (SIEU), reserved memory size, tolerances, results to be written, etc. After that, all the mesh and material data are read, including the arrays *Rmap* and *Ad* (see Appendix B), which were previously generated. The next step is the distribution of random tensile strength, involving the determination of the array *Str* (Algorithm A1). Lines 5–24 basically correspond to the integration of the iterative procedure for nonlinear analysis with Algorithms A2 and A3 at all the load steps. In line 8, the global stiffness matrix is calculated only for *n* = 1 because, for *n* > 1, this matrix will have already been updated in line 19 at the (*n*−1)-th step. If the classical strategy of mesh formation is being used, lines 16 and 17 are not accessed. The solution of the linear system of equations is obtained by using the direct Intel^®^ MKL PARDISO solver.

**Algorithm A5.** Overview of the developed computational program
1. *Read the configuration information*; 2. *Read the mesh and material data*; 3. *Algorithm A1*; 4. n= 0; *// load step counter* 5. **do** (*until the last load step*) 6. n= *n +* 1; 7. i= 0; *// iteration counter* 8. **if** n= 1 **then** *calculate the global stiffness matrix*; 9. *Determine the load increment*; 10. **do** (*until the balance between external and internal forces*) 11. i= *i +* 1; 12. *Compute the linear solution displacements*; 13. *Update the total displacements*; 14. *Update the damage state of the interface elements present in the mesh*; 15. **if** SIEU = AMS1 or SIEU = AMS2 or SIEU = AMS3 **then** 16. *Algorithm A2*; 17. *Algorithm A3*; 18. **end** 19. *Update the global stiffness matrix*; 20. *Calculate the residual force vector*; 21. **end** 22. *Write the load step results*; 23. *Verify the simulation stopping criterion (see Section 3.3)*; 24. **end**

Line 9 of Algorithm A5 refers to the determination of the increment size related to the failure of the interfacial region whose tensile strength is closest to being reached. The procedure for doing this can be called the most dangerous element method. It is important to carry out this step to favor proper stress redistributions during the cracking process as well as to avoid any dependence of the results on the load increment size. The procedure consists in performing a linear analysis with a given load increment, generating a stress increment ∆σ at the interfacial regions. For each intact interfacial region (containing, or not, an interface element), the virtual cracking factor (α) is calculated:

(A1) $$\alpha =\frac{f_{t}^{int}-\sigma_{0}^{t}}{\sigma_{t}-\sigma_{0}^{t}}=\frac{f_{t}^{int}-\sigma_{0}^{t}}{∆\sigma }\;$$

with $\sigma_{0}^{t}$ and $\sigma_{t}$ denoting the initial and final normal tensile stresses, respectively, and α>0. When the tensile strength is not reached, the result is α>1. Hence, this parameter is only relevant when 0<α≤1, indicating that $\sigma_{t}\geq f_{t}^{int}$ in such a way that the lower the value of α, the more imminent is the failure of the interfacial region. After calculating all the relevant values of α, the lowest one represents the percentage of the initial load increment that is actually to be applied. If α is greater than 1 for all interfacial regions, the analysis continues with the initially applied load increment.

In line 14 of Algorithm A5, the terms of the array *Savg* (see Table A1) corresponding to the interfacial regions with interface elements are calculated. If no adaptive mesh strategy is being used, which indicates the use of the classical strategy of mesh formation, the entire process of detecting new cracks and the transition from intact to cracked interface elements occur in this line. In addition, the frictional contact between crack surfaces is evaluated, with the possibility of reactivating or nullifying the rigidity of the cracked interface elements, if necessary.
